# Pruritus Ani

**Published:** 1898-03

**Authors:** 


### PRURITUS ANI.

MATHEWS says that pruritus ani is the most intractable of all the diseases of the anus or rectum, and deserving of our earnest consideration and attention, and I am sure that those of you who have seen this disease will most heartily concur in that statement. It is absolutely necessary to remove the exciting cause or causes in these cases to make a permanent cure. We grant in the outset that this cannot always be accomplished, but these are decidedly the exceptions rather than the rule. Among the exciting causes that are local we will mention pediculi, eczema, erythema (in fat people), thread worms (which are to be found in the radiating folds at the margin of the anus), lack of cleanliness, the disease known as eczema marginatum (which is most easily cured by rubbing well into the parts night and morning for a week or so an ointment containing from ten to thirty grains of chrysophanic acid to the ounce of vaseline), hemorrhoids, fistula, fissures and the like. Some other causes that we would consider as reflex or constitutional are stone in the bladder, chronic inflammation of the deep urethra, stricture of the urethra, pelvic tumors, uterine derangements, functional disorders of the liver, diabetes, constipation, and lastly, but not least, gastro-intestinal disorders, especially that form that is so commonly known as atonic dyspepsia superinduced by smoking, drinking, irregular eating, irregular sleeping; in fact, you might say irregularity in all the walks of life. The treatment which tends to the best results is a light breakfast, no luncheon, a good dinner, plenty of hot water an hour before and between meals and following the homely road of correct habits. Many and innumerable are the local and constitutional remedies we have for the relief of this affection, none of which will I burden you with, but refer you to the text-books on the subject. We do not wish to be thrown out upon the broad field of empiricism or be known as a routinist when we suggest one method which is conducive to the best results in all these cases where no exciting cause or causes remain, that of stretching the rectum under anesthesia for from three to five minutes. After this take a sharp curette and simply remove every vestige of the thickened and parchment-like membrane. When this will have been done you will have a complete change in the condition of the parts; you will have converted a chronic inflammation into that of an acute, whose tendency is always to recovery. You will have removed the contraction of the sphincter muscle, which will admit of a freer circulation of blood through the parts, and taken off entirely the pressure on the terminal filaments of the nerves.—*Maryland Medical Journal.*

---

### AN OINTMENT FOR PRURITUS.

| | | | |
|---|---|---|---|
| ℞ | Mentholi | 4 | (ʒi.) |
| | Cerat. simplicis | 64 | (℥ii.) |
| | Ol. amygdal dulcis | 32 | (℥i.) |
| | Ac. carbolici | 4 | (ʒi.) |
| | Pulv. zinci oxidi | 8 | (ʒii.) |

M. Sig.—Apply morning, noon and night, after cleaning the parts. —*Dr. C. B. Kelsey.*

